# Impact of Health Portal Enrollment With Email Reminders on Adherence to Clinic Appointments: A Pilot Study

**DOI:** 10.2196/jmir.1702

**Published:** 2011-05-26

**Authors:** Monica Horvath, Janet Levy, Pete L'Engle, Boyd Carlson, Asif Ahmad, Jeffrey Ferranti

**Affiliations:** ^3^Department of PediatricsDuke University School of MedicineDurham, NCUnited States; ^2^Duke University School of NursingDurham, NCUnited States; ^1^Duke Health Technology SolutionsDuke University Health SystemDurham, NCUnited States

**Keywords:** Reminder systems, Health Information Technology for Economic and Clinical Health Act, medical informatics applications

## Abstract

**Background:**

Internet portal technologies that provide access to portions of electronic health records have the potential to revolutionize patients’ involvement in their care. However, relatively few descriptions of the demographic characteristics of portal enrollees or of the effects of portal technology on quality outcomes exist. This study examined data from patients who attended one of seven Duke Medicine clinics and who were offered the option of enrolling in and using the Duke Medicine HealthView portal (HVP). The HVP allows patients to manage details of their appointment scheduling and provides automated email appointment reminders in addition to the telephone and mail reminders that all patients receive.

**Objective:**

Our objective was to test whether portal enrollment with an email reminder functionality is significantly related to decreases in rates of appointment “no-shows,” which are known to impair clinic operational efficiency.

**Methods:**

Appointment activity during a 1-year period was examined for all patients attending one of seven Duke Medicine clinics. Patients were categorized as portal enrollees or as nonusers either by their status at time of appointment or at the end of the 1-year period. Demographic characteristics and no-show rates among these groups were compared. A binomial logistic regression model was constructed to measure the adjusted impact of HVP enrollment on no-show rates, given confounding factors. To demonstrate the effect of HVP use over time, monthly no-show rates were calculated for patient appointment keeping and contrasted between preportal and postportal deployment periods.

**Results:**

Across seven clinics, 58,942 patients, 15.7% (9239/58,942) of whom were portal enrollees, scheduled 198,199 appointments with an overall no-show rate of 9.9% (19,668/198,199). We found that HVP enrollees were significantly more likely to be female, white, and privately insured compared with nonusers. Bivariate no-show rate differences between portal enrollment groups varied widely according to patient- and appointment-level attributes. Large reductions in no-show rates were seen among historically disadvantaged groups: Medicaid holders (OR = 2.04 for nonuser/enrollee, 5.6% difference, *P* < .001), uninsured patients (OR = 2.60, 12.8% difference, *P* < .001), and black patients (OR = 2.13, 8.0% difference, *P* < .001). After fitting a binomial logistic regression model for the outcome of appointment arrival, the adjusted odds of arrival increased 39.0% for portal enrollees relative to nonusers (OR = 1.39, 95% CI 1.22 - 1.57, *P* < .001). Analysis of monthly no-show rates over 2 years demonstrated that patients who registered for portal access and received three reminders of upcoming appointments (email, phone, and mail) had a 2.0% no-show rate reduction (*P* < .001), whereas patients who did not enroll and only received traditional phone and mail reminders saw no such reduction (*P* < .09).

**Conclusions:**

Monthly no-show rates across all seven Duke Medicine clinics were significantly reduced among patients who registered for portal use, suggesting that in combination with an email reminder feature, this technology may have an important and beneficial effect on clinic operations.

## Introduction

As the availability of the Internet continues to expand and health care consumers grow increasingly comfortable obtaining information online, surveys show that patients desire access to their personal health care information [1]. Patients have also reported altering their health care behavior based on information they find on the Internet [2]. Given these developments, health portals—novel Web-based applications that allow patients to securely and privately review portions of their electronic health record (EHR), schedule appointments, find educational information, review medications, and even send messages to providers—have the potential to revolutionize patients’ involvement in their own care.

Recently, the Health Information Technology for Economic and Clinical Health **(**HITECH) act (July 2010) defined a detailed program through which Medicaid and Medicare providers could receive incentives for deploying and demonstrating “meaningful use” of certified EHRs [3]. This program may result in increased patient portal use, as one measure of meaningful use requires that more than 50% of patients requesting an electronic copy of their medical record receive access to that information within 3 business days. Portal applications afford an attractive means for meeting this objective, especially given the fact that patients have been shown to frequently use these tools to access aftercare summaries [4].

Reports of portal usage and enrollee demographics have been sporadically published over the past few years; most indicate patients primarily use portals for viewing lab and radiology results [4-6]. The majority of portals described in the literature are health system-dependent, meaning patients can only view their EHR as stored by a single health network. Examination of these studies’ demographic characteristics shows that portal enrollees are more likely to be middle-aged, female, privately insured patients with a higher degree of morbidities [4,7]. These studies typically describe portal deployment and report characteristics of early adopters of the technology, including Kaiser Permanente, the Veterans Affairs health system, Group Health, and Beth Israel Deaconess Medical Center. Meanwhile, there is a notable paucity of follow-up reports describing whether enrollee populations broaden and how portal activities change over time. This absence calls for additional research to assess portal impact on patient outcomes, understand how patients use them, and define a business case for wider adoption [8-11].

Because portals provide patients with direct access to appointment scheduling details, their use may reduce missed appointments—a known barrier to clinic efficiency [12,13] that occurs in 10% to 30% of all appointments [14]. This study describes the demographic characteristics of patients enrolled in the Duke Medicine HealthView portal (HVP) and investigates how portal enrollment may influence appointment attendance at seven Duke Medicine clinics over a 1-year period. Specifically, we sought to test whether portal enrollment coupled with an email appointment reminder function is significantly related to decreases in rates of appointment “no-shows.”

## Methods

### The Duke Medicine HealthView Portal (HVP)

**Figure 1:**
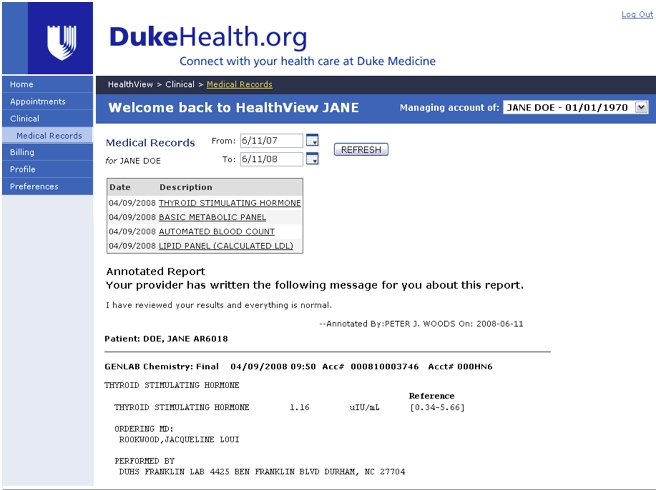
Screenshot of the DUHS HealthView portal

The HVP (deployed in February 2007) is internally developed and supported by a full-time team from Duke Health Technology Solutions (DHTS), an entity within the Duke University Health System (DUHS) that is responsible for information technology initiatives and supports nearly 20,000 full-time health system employees. The HVP is a secure website constructed using IBM WebSphere Portal Server Architecture. The development team includes a manager, two business analysts, a technical support representative, two application system administrators, and three programmers. Additional staff from the DHTS Infrastructure and Operations teams provide ongoing hardware, networking, storage, and database support. The HVP is tested to ensure compatibility with Windows, Macintosh, and Linux operating systems running Firefox, Internet Explorer, and Safari browsers. HVP registration requires patients to provide an active email address and select a password. The HVP website is available outside of the DUHS firewall and, at the time of this study, affords access to appointment and billing information and clinical data pertaining to service at any outpatient Duke Medicine facility (Figure 1). Parents or legal guardians may link children to their own HVP accounts and act as surrogate users. By selecting Medical Records, patients are able to see their laboratory and radiology test results, which may be annotated by physicians. Other functions include viewing and scheduling appointments, reviewing accounts, editing personal and insurance profiles, selecting a preferred method for communication, and managing account settings such as password, email address, and required security questions. All HVP enrollees receive an email reminder 1 week prior to a scheduled appointment as well as an automated telephone call and a physical letter by mail. Nonusers receive only the telephone and mail reminders of upcoming appointments.

### Setting and Patient Population

This study included all DUHS patients with a scheduled appointment in the period from January 1, 2008, through December 31, 2008, at one of seven study clinics located in Durham County, North Carolina: three primary care clinics, three children’s primary care clinics, and one specialty clinic. Clinics were selected based on overall appointment volume and HVP enrollee penetrance. The three primary care clinics were affiliated with Duke Primary Care (DPC), which offers full-service family medicine, general internal medicine, and pediatric medicine practices. Approximately 2600 patients attend 7800 appointments each month under the service of 29 physicians, 26 residents, 9 physician assistants, 2 nurse practitioners, and 2 pharmacists. The three Duke Children's Primary Care (DCPC) clinics provide comprehensive care for newborns, children, and adolescents. Approximately 1300 patients attend 4500 appointments each month under the service of 77 pediatric-focused physicians, 6 physician assistants, and 5 nurse practitioners. We also included one specialty clinic focused on hematology/oncology, as anecdotal evidence in the literature suggests that this group of patients is particularly interested in monitoring their clinical information online [15]. This clinic diagnoses, treats, and helps patients manage solid tumors, lymphomas, and a variety of complex bleeding and clotting disorders. The clinic practice comprises 9 physicians: 6 focused on hematology and 3 on oncology. Approximately 650 patients attend a total of 2400 appointments each month.

### Study Design and Data Collection

We used a retrospective cross-sectional study to assess (1) demographic profiles of HVP enrollees and nonusers and (2) the relationship between HVP enrollment and appointment no-show rates where no-show rates are calculated as the percentage of all arrived and no-show appointments in total that were no-show appointments. 

For direct demographic comparisons between groups, patients were classified as *enrollees* or *nonusers* according to HVP registration status as of December 31, 2008. Patient-level data extracted from the organizational data warehouse included age, patient-reported race, patient-reported ethnicity, sex, and HVP registration date. Patient age was classified into one of six groups and considered as categorical (not continuous) data, as this modeling better fit the observed variability of overall appointment scheduling by age.

For no-show rate comparisons, all 2008 appointment-level data (appointment date, location, visit type, and payor) for the seven study clinics were extracted and classified as *arrived* or *no-show*, indicating whether an appointment was kept, and as *enrollee appointmen*t or *nonuser appointment*, indicating whether the patient had a portal registration prior to the appointment date. “Payor” was defined as the payor identified by the patient at the time of the appointment scheduling and subsequently recorded in our data warehouse (the ultimate party who paid the appointment expenses could potentially differ from the payor identified by the patient). For a secondary analysis, we also collected all 2006 appointments from these seven clinics for the same cohort of patients in order to measure no-show rates among the same cohort prior to HVP deployment.

### Statistical Methods

We tested for significant differences in demographic profiles between portal enrollees and nonusers using chi-square tests for categorical data and nonparametric Wilcoxon rank-sum tests for continuous data. *P* values less than or equal to .05 were considered statistically significant. For clinic appointments scheduled in 2008, no-show rates were compared between enrollees and nonusers and further stratified by patient- and appointment-level characteristics. Odds ratios (ORs) and 95% confidence intervals (CIs) were calculated as the odds of no-show among nonusers divided by the odds of no-show among HVP enrollees. All analyses were performed using JMP 8.0 (SAS Corporation, Cary, NC).

Multiple binomial logistic regression was used to describe the effect of HVP registration with an email appointment reminder feature on 2008 appointment arrivals (coded as 1 = arrived, 0 = no-show) while adjusting for other recognized confounders that could affect this outcome. All modeling was performed in SAS 9.1.3 (SAS Corporation, Cary, NC). An initial model was created using categorical stratification variables deemed relevant to appointment arrival based on ORs from the previous bivariate analysis: HVP registration, appointment clinic, race, age bracket, appointment type, payor, sex, and ethnicity. All variables, as well as their first-order interactions with HVP registration, were entered into the model with reference coding using the PROC LOGISTIC procedure. Each possible predictor variable had multiple levels. Levels within the same variable that had significant overlap in the CIs of the ORs for the no-show appointment outcome were collapsed. The backwards elimination method was used to remove nonsignificant predictor variables and interactions (Wald χ^2^ test, *P* > .05). This defined the most parsimonious model describing appointment arrival. The -2 log likelihood ratio test was used to test the overall model significance, and the Wald χ^2^ tests were used to assess the significance of the predictor variables.

Monthly no-show rates were calculated and plotted over time for clinic appointments from January to December 2006 (prior to HVP deployment) and then again from January to December 2008. Monthly no-show rates were subdivided by appointments for HVP enrollees versus nonusers. Only those patients who had at least one appointment in both 2006 and 2008 were included. Error bars were calculated as the 95% CI of each month’s no-show rate. Monthly no-show rates between compared groups were matched by month and the paired *t* test was used to assess statistical significance.

## Results

### Characteristics of HealthView Portal Enrollees

Across our seven study clinics, 58,942 patients scheduled a total of 198,119 appointments in 2008. A total of 13,265 patients failed to arrive for 19,668 appointments, resulting in an overall no-show rate of 9.9%. Of all 58,942 patients, 2838 (4.8%) were registered for HVP use at the beginning of the study, and an additional 9239 patients (15.7%) had registered by December 31, 2008. Consistent with previous reports [4-7,11], the 12,077 portal enrollees were significantly more likely to be female (63.8%, 7702/12,077), white (70.3%, 8495/12,077), to hold private health insurance (83.5%, 10,080/12,077), and to be between 40 and 65 years old (52.6%, 6,354/12,077) (Table 1, all *P* < .001). Portal enrollees in the 17 years of age and under age group comprised only 4.7% (563/12,077) of the enrollee population but 39.1% (18,345/46,865) of the nonuser population, likely because children usually have portal access through a parental surrogate. Both enrollees and nonusers had a median of two scheduled appointments per year, although nonusers scheduled more appointments. Nearly all comparisons of demographic factors between enrollees and nonusers were statistically significant with the exception of the proportion of individuals in the age group 18 to 29 years (*P* = .75) and the age group 65 years of age and older (*P* = .88).

**Table 1:** Demographic characteristics of patients seen at seven Duke Medicine clinics during 2008

		All Patients (n = 58,942)	HVP Enrollees^a^ (n = 12,077)	Nonusers^a^ (n = 46,865)	*P*^b^
		n (%)	n (%)	n (%)	
Female		34,094 (57.8)	7702 (63.8)	26,392 (56.3)	< .001
**Age group**					
	≥ 65	8569 (14.5)	1750 (14.5)	6819 (14.6)	.88
	55-64	7441 (12.6)	2536 (21.0)	4905 (10.5)	< .001
	40-54	11,619 (19.7)	3818 (31.6)	7801 (16.6)	< .001
	30-39	6601 (11.2)	2211 (18.3)	4390 (9.4)	< .001
	18-29	5804 (9.8)	1199 (9.9)	4605 (9.8)	.75
	≤ 17	18,908 (32.1)	563 (4.7)	18,345 (39.1)	< .001
**Ethnicity**					
	Hispanic/Latino	2263 (3.8)	147 (1.2)	2116 (4.5)	< .001
	Other	56,679 (96.2)	11,930 (98.8)	44,749 (95.5)	< .001
**Race**					
	White	29,621 (50.2)	8495 (70.3)	21,126 (45.1)	< .001
	Black	22,182 (37.6)	2415 (20.0)	19,767 (42.2)	< .001
	Asian	2135 (3.6)	557 (4.6)	1578 (3.4)	< .001
	Other	5004 (8.5)	610 (5.1)	4394 (9.4)	< .001
**Payor class**^c^					
	Medicaid	9888 (16.8)	199 (1.6)	9689 (20.7)	< .001
	Medicare	8301 (14.1)	1539 (12.7)	6762 (14.4)	< .001
	Private	39,243 (66.6)	10,080 (83.5)	29,163 (62.2)	< .001
	Uninsured/self-pay	848 (1.4)	63 (0.5)	785 (1.7)	< .001
	Unknown	662 (1.1)	196 (1.6)	466 (1.0)	< .001

^a^ Enrollee status as of 12/31/2008

^b^ *P* value by 2-tailed χ^2^ test

^c^Payor as of last 2008 appointment

### Analysis of Appointment No-Show Rates Across User Groups

Table 2 shows appointment no-show rates in both portal enrollees and nonusers during the study period. For an appointment to be classed as an enrollee appointment, the patient must have registered for an HVP account before the scheduled appointment date. In this way, patients who became enrollees over the course of the study could still have their appointment arrival activity appropriately categorized and analyzed. The unadjusted ORs describe the odds of a nonuser failing to keep a scheduled appointment relative to the odds of an enrollee failing to keep a scheduled appointment. Overall, nonusers had 2.26 times the odds of missing a scheduled appointment relative to enrollees receiving email reminders (*P* < .001). Although male nonusers had higher no-show rates than female nonusers, male enrollees had lower no-show rates than female enrollees, suggesting that HVP use may specifically improve appointment keeping in this subgroup. All age brackets had statistically significant no-show rates between enrollees and nonusers, but the greatest reduction in no-show rates was observed in the age group 18 to 29 years (OR = 2.35, rate difference of 8.11), whereas a more modest difference in no-show rates was observed among those in the age group 65 years and over (OR = 1.74).

Although all comparisons were statistically significant, uninsured/self-pay patients and Medicaid holders displayed the largest between-group difference in payor-stratified no-show rates (ORs 2.60 and 2.04, respectively), which represents a no-show rate difference between enrollee groups of 9.0% to 12.7%. This is a much greater difference than was seen between other payor types such as private insurance or Medicare (ORs 1.62 and 1.79, respectively), which represents a statistically different and yet much smaller no-show rate difference of 2.8% to 3.1% between enrollee groups. This pattern remained when race was examined, as black patients (a historically disadvantaged group) had an OR of 2.14 (no-show rate difference between groups of 8.0%, *P* < .001), whereas the OR calculation in white patients was 1.42 (no-show rate difference between groups of 1.6%, *P* < .001). Asians were the only group that exhibited a statistically similar no-show rate between enrollees and nonusers (4.1% and 5.5%, *P* = .09).

In terms of appointment-level characteristics, the hematology/oncology clinic had the lowest OR (1.58) for nonusers versus enrollees, a finding consistent with the fact that these patients are chronically ill and are thus more likely than the primary care groups to keep their appointments. When scheduled appointments were examined by type, consults (OR = 2.77) and new visits (OR = 2.53) had the largest no-show rate differences among visit classes. The day of week was also examined. Interestingly, there was an 8.8% difference in no-show rates between nonusers and enrollees on Saturdays (OR = 3.83), but only a 5.3% to 5.8% difference on other days (OR range 1.95 - 2.37). Taken together, these data suggest appointments outside of a patient’s normal personal schedule have a higher chance of arrival relative to other appointment types if registration for the HVP exists and an email reminder of upcoming appointments is sent.

**Table 2:** No-show rates by portal enrollee status at time of clinic appointment

Parameter		Nonuser No-Show Rate (%)	HPV Enrollee No-Show Rate (%)	*P*^a^	OR Nonuser/ Enrollee (95% CI)
All appointments		10.6 (18,423/173,204)	5.0 (1245/24,915)	< .001	2.26 (2.2-2.33)
**Race**					
	Asian	5.5 (277/5013)	4.1 (37/900)	.09	1.36 (1.14-1.63)
	Black	16.4 (12,325/75,141)	8.4 (418/4949)	< .001	2.13 (2.02-2.24)
	White	5.7 (4468/78,317)	4.1 (745/18,209)	< .001	1.42 (1.36-1.48)
	Other	9.2 (1353/14,733)	5.3 (45/857)	< .001	1.82 (1.56-2.13)
**Age group**					
	≥ 65	5.9 (1718/29,182)	3.5 (169/4862)	< .001	1.74 (1.6-1.89)
	55-64	6.8 (1360/19,964)	4.1 (236/5701)	< .001	1.69 (1.57-1.82)
	40-54	8.6 (2391/27,829)	5.2 (378/7238)	< .001	1.71 (1.61-1.81)
	30-39	10.6 (1526/14,372)	5.5 (196/3537)	< .001	2.02 (1.87-2.19)
	18-29	15.2 (2311/15,203)	7.1 (150/2117)	< .001	2.35 (2.15-2.57)
	≤ 17	13.7 (9117/66,654)	8.0 (116/1460)	< .001	1.84 (1.67-2.02)
**Sex**					
	Male	10.8 (7988/73,781)	4.7 (436/9184)	< .001	2.44 (2.32-2.56)
	Female	10.5 (10,435/99,423)	5.1 (809/15,731)	< .001	2.16 (2.08-2.25)
**Ethnicity**					
	Hispanic/Latino	10.4 (946/9128)	6.7 (20/299)	.04	1.61 (1.28-2.04)
	Other	10.7 (17,477/164,076)	5.0 (1225/24,616)	< .001	2.28 (2.21-2.35)
**Day of week**					
	Monday	11.0 (4012/36,611)	5.2 (283/5441)	< .001	2.24 (2.11-2.39)
	Tuesday	10.2 (3712/36,564)	4.9 (291/5988)	< .001	2.21 (2.08-2.35)
	Wednesday	10.6 (3663/34,667)	4.8 (225/4705)	< .001	2.35 (2.19-2.52)
	Thursday	10.1 (3299/32,747)	4.5 (214/4738)	< .001	2.37 (2.20-2.55)
	Friday	11.4 (3454/30,169)	5.8 (228/3922)	< .001	2.09 (1.95-2.25)
	Saturday	11.6 (283/2446)	3.3 (4/121)	.003	3.83 (2.29-6.39)
**Insurance**					
	Medicaid	19.0 (7926/41,689)	10.3 (76/737)	< .001	2.04 (1.81-2.31)
	Medicare	7.2 (2166/30,165)	4.1 (182/4402)	< .001	1.79 (1.66-1.94)
	Private	7.7 (7440/96,687)	4.9 (937/19,150)	< .001	1.62 (1.56-1.68)
	Uninsured/self-pay	23.2 (586/2525)	10.4 (17/163)	< .001	2.60 (2.00-3.37)
	Unknown	14.3 (305/2138)	7.1 (33/463)	< .001	2.17 (1.79-2.62)
**Visit type**					
	Return visits	10.3 (15,509/149,974)	4.6 (964/20,904)	< .001	2.39 (2.31-2.47)
	Study visits	12.7 (1970/15,541)	7.5 (236/3133)	< .001	1.78 (1.66-1.91)
	Consults	13.5 (343/2539)	5.3 (20/375)	< .001	2.77 (2.19-3.51)
	New visits	11.7 (601/5150)	5.0 (25/503)	< .001	2.53 (2.05-3.12)

**Clinic**					
	Duke Children’s Primary Care, clinic 1	6.1 (878/14,349)	5.4 (33/615)	.49	1.15 (0.96-1.38)
	Duke Children’s Primary Care, clinic 2	16.4 (7360/45,022)	10.4 (122/1169)	< .001	1.68 (1.52-1.85)
	Duke Children’s Primary Care, clinic 3	16.4 (465/2839)	2.3 (1/43)	.01	8.23 (2.99-22.66)
	Duke Primary Care, clinic 1	8.2 (976/11,953)	5.4 (353/6542)	< .001	1.56 (1.46-1.66)
	Duke Primary Care, clinic 2	13.1 (5102/38,817)	5.4 (307/5666)	< .001	2.64 (2.49-2.81)
	Duke Primary Care, clinic 3	4.5 (1525/34,031)	2.6 (130/5086)	< .001	1.79 (1.63-1.96)
	Hematology/oncology clinic	8.2 (2158/26,193)	5.3 (312/5794)	< .001	1.58 (1.49-1.68)

^a^ *P* value by 2-tailed χ^2^ test

### Assessment of Portal Impact After Logistic Regression Modeling

In order to better understand the effect of HVP registration on appointment arrivals, a logistic regression model was created to obtain ORs adjusted for confounders that may affect this relationship. Table 3 shows the maximum likelihood estimates for the parameters of which the most parsimonious model is composed. The overall model was considered significant compared with the null hypothesis, which states the parameters have slopes of zero (-2 log likelihood = 128,176.7, *P* < .001*)*. This model retains a significant term for HVP enrollment (*P* < .001, OR = 1.39, 95% CI 1.22-1.57) after the addition of other parameters and backwards elimination. We obtained a better model by identifying each of the seven clinics uniquely in the model as opposed to grouping them into classes as in Table 2. Only the interaction terms between HVP enrollee status and clinic remained significant after backward elimination. From these data, we conclude the adjusted odds of appointment arrival are increased 39.0% for portal enrollees over nonusers. Compared with the unadjusted OR (2.26), the decrease in the HVP OR in the adjusted model (OR = 1.39) captures the confounding effects the other predictors have on the relationship between HVP enrollment and appointment arrival.

**Table 3:** Multivariable logistic regression model for the outcome of arrived appointment

Variable		Coefficient (Beta)	SE Beta	Wald χ^2^	*P*	OR	95% CI
Intercept		2.97	0.07	2009.7	< .001		
**HVP status**							
	Nonuser (reference)						
	Enrollee	0.33	0.07	25.1	< .001	1.39	1.22-1.57
**Clinic**							
	Duke Primary Care, clinic 1 (reference)						
	Duke Primary Care, clinic 2	−0.12	0.04	9.2	.003	0.89	0.82-0.96
	Duke Primary Care, clinic 3	0.80	0.04	328.7	< .001	2.22	2.04-2.42
	Duke Children’s Primary Care, clinic 1	0.31	0.06	27.9	< .001	1.36	1.21-1.52
	Duke Children’s Primary Care, clinic 2	−0.25	0.05	28.2	< .001	0.78	0.71-0.85
	Duke Children’s Primary Care, clinic 3	−0.16	0.07	5.5	.02	0.85	0.74-0.97
	Hematology/oncology clinic	0.07	0.04	3.1	.08	1.08	0.99-1.17
**Sex**							
	Male (reference)						
	Female	0.06	0.01	14.4	< .001	1.06	1.03-1.10
**Race**							
	White (reference)						
	Asian	0.09	0.06	2.3	.13	1.10	0.97-1.24
	Black	−0.82	0.02	1798.2	< .001	0.44	0.42-0.46
	Other	−0.21	0.05	23.1	< .001	0.80	0.73-0.88
**Age**							
	40-64 (reference)						
	≤ 17	0.01	0.04	0.3	.60	1.02	0.95-1.09
	18-29	−0.35	0.03	131.2	< .001	0.70	0.66-0.75
	30-39	−0.25	0.03	65.8	<.001	0.78	0.73-0.83
	≥ 65	0.43	0.04	133.5	< .001	1.54	1.43-1.66
**Appointment type**							
	Return visits (reference)						
	Consults	0.09	0.06	2.4	.12	1.10	0.98-1.23
	New visits	−0.18	0.05	16.0	< .001	0.83	0.76-0.91
	Clinical trial visits	−0.51	0.03	389.7	< .001	0.60	0.57-0.63
**Ethnicity**							
	Hispanic (reference)						
	Other	−0.18	0.05	11.5	< .001	0.84	0.75-0.93
**Payor**							
	Private insurance (reference)						
	Medicaid/indigent	−0.53	0.02	632.6	< .001	0.59	0.56-0.61
	Medicare	−0.22	0.03	40.8	< .001	0.80	0.75-0.86
	Uninsured	−0.93	0.05	354.9	< .001	0.39	0.36-0.43
	Unknown	−0.28	0.06	20.5	< .001	0.76	0.67-0.85

Interaction with HVP enrollee (Duke Primary Care, clinic 1*HVP enrollee)							
	Duke Primary Care - clinic 2	−0.21	0.20	1.2	.27	0.81	0.55-1.18
	Duke Primary Care - clinic 3	−0.11	0.11	0.8	.37	0.90	0.71-1.13
	Duke Children’s Primary Care - clinic 1	1.40	1.02	1.9	.17	4.03	0.55-29.70
	Duke Children’s Primary Care - clinic 2	0.31	0.09	11.7	< .001	1.36	1.14-1.62
	Duke Children’s Primary Care- clinic 3	0.13	0.11	1.3	.26	1.14	0.91-1.42
	Hematology/oncology clinic	−0.05	0.09	0.3	.61	0.95	0.80-1.14

### Analysis of No-Show Rates Over Time

**Figure 2:**
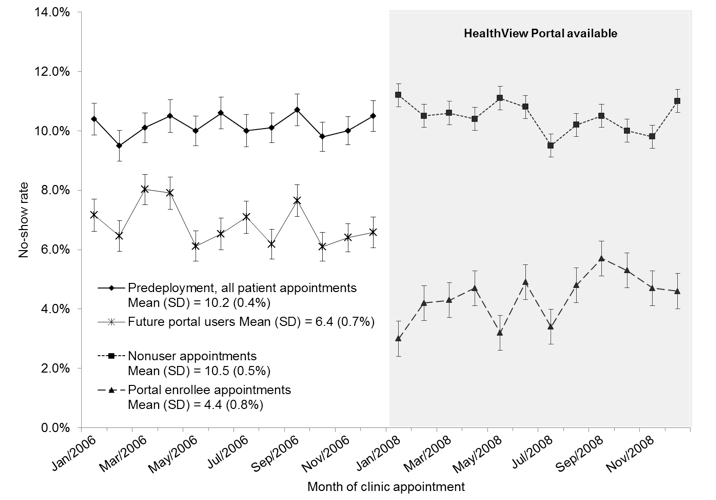
Monthly appointment no-show rates over time for seven DUHS clinics

In order to understand how patient no-show rates changed over time in this study, we examined the monthly trend in no-show rates prior to HVP deployment. Shown in Figure 2 are monthly appointment no-show rates over time for the seven DUHS clinics. All patients that had at least 1 appointment in both 2006 (pre-HVP deployment) and 2008 (HVP fully deployed as of March 2008) were included in the graph. Of the original 58,942 patients, 37,408 had 158,420 scheduled appointments at the seven study clinics during 2006. Of these, 35.8% (13,407/37,408) had 1 or more missed appointments in either 2006 or 2008. In the predeployment period, patients had an average monthly no-show rate of 10.2 (SD 0.4%) (Figure 2). In the postdeployment period (2008), appointments were broken out by HVP registration status. The 2008 nonuser group had a mean monthly no-show rate of 10.5 (SD 0.5%), which was statistically indistinguishable from the mean monthly no-show rate prior to HVP deployment (*P* *=* .09). Relative to the 2006 predeployment phase, no-show rates among the HVP enrollee group fell to a monthly mean of 4.4 (SD 0.8%) (*P* *<* .001). These results show that the HVP enrollee group uniquely improved its appointment attendance rate. The mean no-show rate for patients in 2006 who would become enrollees by the end of the study was 6.4 (SD 0.7%), 2.0% greater than the no-show rate for appointments scheduled by these same patients and clinics in 2008 (*P* *<* .001).

## Discussion

### Principal Results and Comparison With Prior Work

In this study, we developed a demographic profile of portal users at Duke Medicine clinics and found that monthly clinic no-show rates were reduced considerably among those patients having registered for portal usage, as they would have received email reminders of upcoming appointments. Portal enrollment was a significant predictor of appointment arrival even after adjustment for confounding factors such as patient demographics.

Although it is well-established that missed appointments can be costly to providers and decrease operational efficiency, there is little information in the literature regarding the effect of health information technology (HIT) applications on scheduled clinic appointment arrivals. Most reports focus on practices outside the United States—a context in which no-show rates vary considerably and the use of reminder methods such as text messaging have been shown to reduce no-show rates by amounts ranging from 0.8% to 14.5% [16-19]. Mailed reminders have been credited with reducing no-show rates by 10% [14]; telephone reminders have been shown to reduce rates by proportions that ranged from 5.8% to as much as 9.5% [20]. In our study, we saw a more modest but still highly significant 5.6% difference in no-show rates, as stratified by portal enrollment. Portal enrollment remains a significant independent predictor of appointment arrival even when adjusted by potential confounders in a multivariable regression model (OR = 1.39). Because all patients in our study received both telephone and mailed reminders, the improved arrival rate attributable to this HIT application may represent the next needed level of patient involvement beyond simpler reminder methods.

In our study, portal enrollees were typically white, female, middle-aged, and held private health insurance, findings consistent with demographic profiles reported by groups developing the PatientSite [6] and MyGroupHealth [4] portals. There is great concern that the overall impact of portal technologies will not transcend socioeconomic lines and age barriers and access will thus be restricted to younger, healthier, and wealthier persons [21]. We, too, see evidence of this “digital divide” based on socioeconomic status, given that Medicaid patients comprise only 1.7% of portal registrants.

Interestingly, the bivariate analysis of no-show rates demonstrates that the greatest improvement in appointment keeping is seen among traditionally disadvantaged populations who do use the portal. The no-show rate of Medicaid recipients and uninsured/self-pay patients fell by 8.7% and 12.8% respectively for portal enrollees relative to nonusers (each *P* < .001) whereas scheduled appointments belonging to Medicare and privately insured patients showed a 2.8% to 3.1% reduction in the no-show rate. Similarly, black patients who registered for the Duke Medicine HVP showed an 8.0% reduction in the rate of no-shows (*P* < .001) compared with a much more modest reduction among white patients (1.6%, *P* < .001).

Taken together, these data suggest that efforts to enroll patients in HVPs should focus on traditionally disadvantaged populations in order to achieve the greatest gains in appointment keeping. A recent report evaluated several cases of personal health record implementation and concluded that while disadvantaged populations tend not to be early adopters of such technology, patients with limited resources do use online health materials [22], a finding that lends further support for our approach. In fact, a survey of more than 17,000 Medicaid beneficiaries from Durham County, NC, found that 52% of beneficiaries had high-speed Internet access and 64.5% of beneficiaries would view health information through a portal at least once a year if given the option [23]. Although the digital divide also describes barriers for seniors, we found that 20.4% of persons aged 65 years of age or older in our study registered for the HVP by the end of 2008—a proportion that accounts for 14.5% of all enrollees and does not differ statistically from the proportion of seniors in the nonuser group (*P* = .89). However, the PatientSite study, using data gathered in 2004, noted that seniors made up only 7% of enrollees. Increasing access to technology over time may help account for these disparate findings.

With the exception of minors (who most often have portal accounts through parental surrogates), the no-show ORs contrasting nonusers with portal enrollees decreased as age increased to 65 years and older. Although more research is needed, we might infer that older persons are more independently active in managing their own health care and thus may benefit less from a portal in terms of its effect on keeping appointments. Similarly, young people may be healthier and possibly less responsible, meaning that portal-generated reminders are proportionately more helpful for keeping appointments.

Individual clinic was an important independent predictor of appointment arrival in the multivariable model; further, the no-show rate differences between enrollee groups varied by clinic class. Only DPC clinic 2 had statistically significant interaction with portal enrollment, indicating portal enrollment is more influential on no-show rates for this clinic relative to the other clinics. The reason for this difference is not clear and may be due to the unique environment of each clinic, differential responses among patient groups to portal marketing efforts, or the technology itself. Compared with the primary care clinics, the hematology/oncology specialty clinic saw the most modest no-show rate difference across enrollee groups (2.9%) despite having the same proportion of portal enrollees as the adult primary care clinics (17% of all appointments were scheduled by HVP enrollees in both groups). Patients with serious long-term illness have more pressing ongoing care needs; it is thus understandable that they have lower no-show rates. The impact of portal technologies across different types of patients may need to be evaluated in terms of different quality-of-care metrics. For example, a recent randomized controlled trial showed that diabetic patients who used an EHR-integrated portal had their treatment regimens adjusted more often than nonusers [24].

Our analysis of monthly no-show rates following HVP deployment illustrates that portal enrollees consistently demonstrate a 6.1% lower rate of appointment no-shows compared with nonusers. However, it is possible that HVP enrollment did not improve patient attendance so much as identify the most compliant and engaged set of patients. Our adjusted odds of appointment arrival for enrollees versus nonusers is 1.39, which is similar to other published regression models evaluating the effect of reminder methods on this outcome (eg, Parikh et al reported adjusted ORs of 1.58 to 1.98 for telephone calls relative to a control group receiving no reminders [20]). Moreover, when no-show rates from the period before deployment in 2006 were examined, rates for those who would later enroll were 2.0% higher across the same set of clinics, indicating the pattern of appointment attendance improved even among patients who were potentially more compliant.

### Limitations

This study has several limitations. Our findings are drawn from only seven clinics within a large health system and describe a period of time when the HVP was a relatively new addition to patient care and thus may not be applicable to all health care settings. Due to the structure of the log files that described patient use of the portal, we were able to capture patient registration dates in order to identify enrollees but were unable to capture enrollee attrition. Ideally, detailed information regarding log-ins and page views would better define the profile of active enrollees and make a stronger case that active HVP use—not just registration and receipt of email appointment reminders—is an important component of patient arrivals. A project is underway to extract information on HVP use from application log files and load those data originating from July of 2009 forward into the organizational data warehouse. At the time of this writing, the HVP currently has more than 100,000 registered enrollees. We expect a future study analyzing enrollee demographics across all DUHS clinics will allow us to better estimate the effect of the technology on patient care management and allow development of a more rigorous predictive model that explains not only appointment arrivals, but also patient care outcomes such as emergency department utilization or long-term disease management. In this future work, we hope to identify patterns of clinic characteristics strongly associated with appointment keeping and portal enrollment, as was seen in this study with DPC clinic 2.

We did not collect data on advance cancellations. Hagerman and colleagues reported that patient reminders might increase the likelihood of cancellations [25], although this trend has not been observed elsewhere [14]. In our study, all patients were notified by telephone and mail. Only HVP enrollees received an additional reminder via email. However, even if an increase in advance cancellations was present in the HVP enrollee arm, this still represents a net benefit to study clinics, because staffing could be adjusted as needed and other patients could be accommodated on short notice. This question will be a focus of future analysis across a wider set of clinics.

Finally, we did not collect data on the projected costs of a no-show appointment. Given that our study was conducted among high-volume clinics served by a large number of providers, the wide variability in providers makes it difficult to determine the specific amount of revenue lost due to no-show appointments. Data on the cost of appointment no-shows are limited, and actual costs are likely to be highly clinic-dependent; thus, the issue of financial impact merits its own separate study. But given the effect of missed appointments on clinic operating costs and efficiency, such a study would help inform the decision of whether Duke Medicine should consider creating incentives for patients to use portal technologies.

### Conclusions

This study developed a demographic profile for enrollees of the Duke Medicine HVP and described the relationship between portal registration and appointment arrival rates when email reminders are in place. As seen in other studies, historically disadvantaged groups are less likely to use the portal, providing further evidence of a digital divide. Monthly no-show rates across seven DUHS clinics were reduced considerably among those patients who registered for portal usage and received email reminders, suggesting this technology may have important beneficial effects on clinic operations, as nonusers would have only received mail and telephone reminders. Portal enrollment was a significant predictor of appointment arrival even when adjusted for known confounders such as payor, clinic, and race. The greatest proportional improvements in appointment attendance associated with portal enrollment were seen among historically disadvantaged groups. Further research that would examine actual log-in activity is needed to understand the relationship between different types of portal usage and more nuanced quality and safety outcomes in order to better elucidate the overall profile of portal effectiveness.

## Abbreviations

CI: confidence interval
DCPC: Duke Children's Primary Care
DHTS: Duke Health Technology Solutions
DPC: Duke Primary Care
DUHS: Duke University Health System
EHR: electronic health record
HIT: health information technology
HITECH: Health Information Technology for Economic and Clinical Health
HVP: HealthView portal
OR: odds ratio
